# Natural Compound-Derived Cytochrome bc1 Complex Inhibitors as Antifungal Agents

**DOI:** 10.3390/molecules25194582

**Published:** 2020-10-07

**Authors:** Loana Musso, Andrea Fabbrini, Sabrina Dallavalle

**Affiliations:** Department of Food, Environmental and Nutritional Sciences (DeFENS), Division of Chemical and Biomolecular Sciences, Università degli Studi di Milano, Via Celoria 2, 20133 Milan, Italy; andrea.fabbrini@unimi.it (A.F.); sabrina.dallavalle@unimi.it (S.D.)

**Keywords:** natural compounds, cytochrome bc1 complex, antifungals, (E)-β-methoxyacrylate, strobilurins

## Abstract

The high incidence of fungal pathogens has become a global issue for crop protection. A promising strategy to control fungal plant infections is based on the use of nature-inspired compounds. The cytochrome bc1 complex is an essential component of the cellular respiratory chain and is one of the most important fungicidal targets. Natural products have played a crucial role in the discovery of cytochrome bc1 inhibitors, as proven by the development of strobilurins, one of the most important classes of crop-protection agents, over the past two decades. In this review, we summarize advances in the exploration of natural product scaffolds for the design and development of new bc1 complex inhibitors. Particular emphasis is given to molecular modeling-based approaches and structure–activity relationship (SAR) studies performed to improve the stability and increase the potency of natural precursors. The collected results highlight the versatility of natural compounds and provide an insight into the potential development of nature-inspired derivatives as antifungal agents.

## 1. Introduction

Plant diseases caused by fungal pathogens are a major threat to the agricultural industry worldwide. Controlling plant fungal infections is therefore key to increase food production, as the planet’s continuously growing population demands maximized crop yields [1].

**Figure 1:**
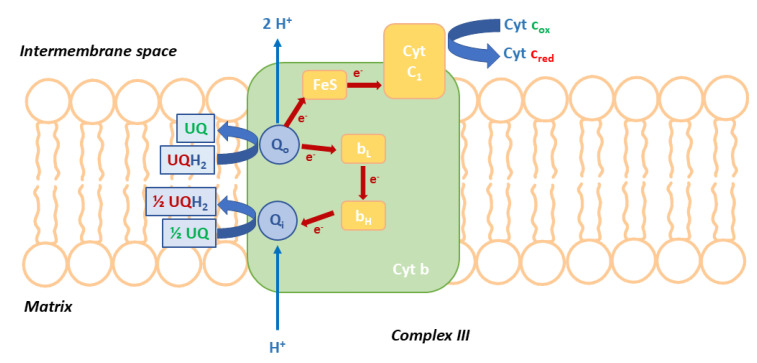
Schematic model of cytochrome bc1 complex. The homodimeric bc1 complex presents three catalytic subunits: cytochrome b (cyt b) with two b-type haems (b_H_ and b_L_), the Rieske iron–sulfur protein (FeS) and cytochrome c1 (cyt c1) with one c-type haem. The two binding sites for inhibitors and ubiquinone (UQ), Qi and Qo, are shown. The bifurcated electron transfer pathway from the Qo site is shown by red arrows.

The cytochrome bc1 complex (also known as complex III) is one of the most important fungicidal targets. The complex is an essential component of the cellular respiratory chain. It catalyzes the ubiquinol oxidation (UQH2) to quinone (UQ) and channels the resulting electrons to cytochrome c through the ubiquinone cycle (Qo cycle, Figure 1). Furthermore, the ubiquinol oxidation results in a bifurcated electron transfer. One electron is transferred, via the Rieske iron–sulfur protein and cytochrome c1, to cytochrome c, while the second electron passes through two b-type haems (b_L_ and b_H_) to reduce ubiquinone at the Qi site [2].

This process results in proton translocation and generates the proton motive force required in the production of ATP. Protons are taken up from the mitochondrial matrix when ubiquinone is reduced at the Qi site and they are released into the intermembrane space when ubiquinol is oxidized at the Qo site.

Inhibition of the activity of the bc1 complex blocks the generation of ATP, leading to cell death. For this reason, inhibitors of the cytochrome bc1 complex have aroused great interest in controlling fungal diseases. The cyt bc1 inhibitors target either the ubiquinol oxidation site (Qo or Q_P_) or the ubiquinone reduction site (Qi or Q_N_).

Natural products have played a crucial role in the investigation of cytochrome bc1 inhibitors, leading to the discovery of one of the most important classes of crop-protection agents, the strobilurins. These compounds still have a dominant position in the global market.

Natural products, with their enormous structural diversity, are an invaluable source of inspiration in the design and development of new biologically active compounds [3]. Having evolved over several millennia to acquire specific ligand–protein binding motifs, these privileged compounds serve as important, biologically pre-validated platforms for the development of new leads in medicinal chemistry and agriculture. However, the structural complexity, toxicity, and unfavorable bioavailability often associated with natural products can limit their potential, which is why as such structural modification is often required.

In this review, we summarize the efforts towards the exploration of natural product scaffolds for the design and development of bc1 complex inhibitors, focusing on what has been observed and achieved in the past decade. In particular, we concentrate on studies focused on optimization of the core natural scaffold by simplification, direct substitution, and/or by use of isosteric modifications. Molecular modeling-based approaches and structure–activity relationship (SAR) studies performed to improve the potency, selectivity, and stability of bioactive natural products are highlighted as well.

## 2. Strobilurins

Strobilurins are natural compounds isolated for the first time in 1977 from basidiomycetes. They are the first example of natural fungicides belonging to the group of QoI (quinone outside inhibitors, (FRAC group 11)). Strobilurin-based products, obtained by activity-guided rational design, have been a milestone in the fungicidal market worldwide. Indeed, these compounds have a broad-spectrum of activity, being effective against all four major classes of phytopathogenic organisms (ascomycetes, basidiomycetes, deuteromycetes, and oomycetes). They show acute toxicity against germinating fungal spores and relatively low toxicity for terrestrial animals.

The major drawbacks of these fungicides are their toxicity to aquatic organisms and the increased number of fungal pathogens that are showing occurrence of resistance, mainly due to mutations on mitochondrial genes encoding for the Qo site [4].

Based on this evidence, the development of new strobilurin derivatives is still pressing because of their role in the market and the need of overcoming the explosive increase of fungal resistance worldwide.

Various reviews have been published about the design and synthesis of new strobilurin-derived compounds [5,6]. In the next section, we provide an overview of the efforts made in this field in the last 10 years.

### From Natural to Synthetic Strobilurins

**Figure 2:**
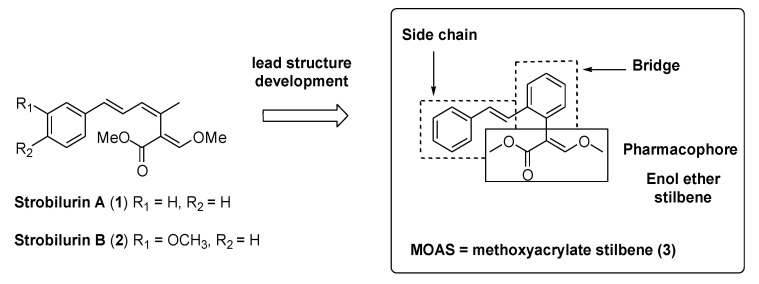
Structure of natural strobilurin A and B, and the methoxyacrylate stilbene (MOAS) lead structure.

Strobilurin A (**1**) was the first compound of this family isolated from *Strobilurus tenacellus* by Anke et al. [7], followed by strobilurin B (**2**) and so on (Figure 2). Although isolated from different fungi, the various strobilurins have a very similar structure characterized by a pharmacophore portion, an unsaturated bridge, and a side chain, varying only in the aromatic ring substituents. The interest for strobilurins increased following the discovery of their fungicidal activity by Anke et al. [7], which led scientists to search for these compounds in various fungi. However, their activity was weak and, more importantly, they exhibited stability problems because of their photo lability.

During the first 1980s, the ICI and the BASF groups simultaneously, although working separately, identified methoxyacrylate stilbene (**3**, MOAS, Figure 2) as a stable synthetic strobilurin with a benzene ring in place of the central double bond of the original triene system [6].

**Figure 3:**
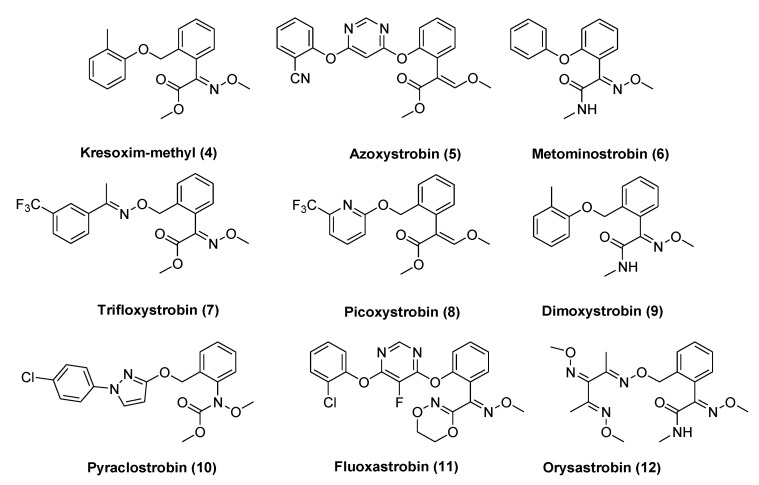
Commercial strobilurins.

Compound **3** immediately became the lead synthetic strobilurin because of its stability and increased efficacy (Figure 2). Successive studies led to the introduction into the market of various synthetic analogs, featuring broad structural variations on the side chain portion as well as containing bioisosteres of the (E)-β-methoxyacrylate pharmacophoric group (Figure 3) [8].

While the early chemical modifications of the strobilurin scaffold addressed the improvement of physical characteristics (i.e., volatility, stability, resistance to UV breakdown) and resulted in commercial strobilurins like azoxystrobin, picoxystrobin, pyraclostrobin, and trifloxystrobin (Figure 3), in the latest years new strobilurins were developed to increase their efficacy as well as to overcome the resistance to their action on QoI-resistant strains.

For this purpose, different approaches were used, such as the me-too approach, biorational or chemorational design, fragment-based drug design (FBDD), intermediate derivatization methods based on bioisosteric replacement, and pharmacophore-linked fragment virtual screening (PFVS).

**Figure 4:**
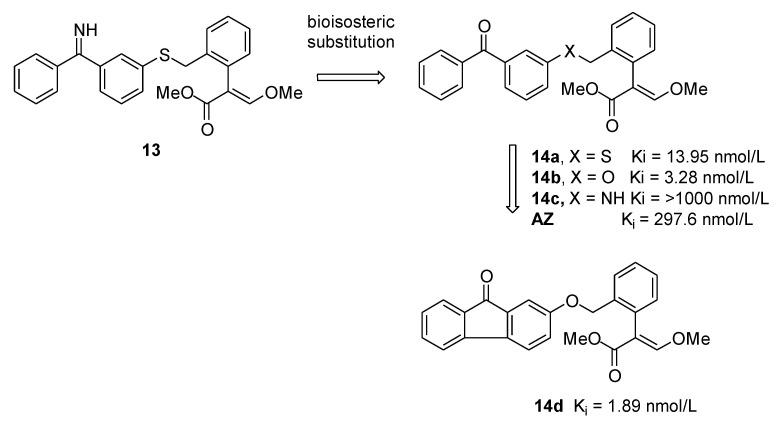
Structural optimization of lead compound **13** and inhibitory activities of representative compounds **14a**–**14d** and azoxystrobin (AZ) against porcine SCR (succinate cytochrome c reductase) with cytochrome c as substrate.

Hao et al. developed a new molecular design method based on PFVS, by integrating the advantages of FBDD and the advantages of docking methods [9]. Through this approach, Yang and coworkers [10] designed and synthesized a series of benzophenone/fluorenone-containing derivatives (Figure 4) to obtain new strobilurin analogs with higher fungicidal activity. As shown in Figure 4, the *O*-bridged derivative **14b** (K_i_ = 3.28 nmol/L) is more active than its corresponding *S*-bridged derivative **14a** (K_i_ = 13.95 nmol/L) and the *NH*-bridged derivative **14c** (K_i_ > 1000 nmol/L). Interestingly, compound **14d** showed an improved binding affinity (K_i_ = 1.89 nmol/L) to the porcine cytochrome bc1 complex (porcine SCR, succinate cytochrome c reductase) compared to the commercial inhibitor azoxystrobin.

The binding mode of compound **14d** in Qo site of bc1 complex demonstrated that the pharmacophore of this new inhibitor bound in the same way of typical methoxyacrylate inhibitors, interacting with Phe128, Tyr131, Phe274, and Glu271, and forming an H-bond between the methoxy group of the methoxyacrylate moiety and the backbone nitrogen of Glu271. The presence of the fluorenone ring in **14d** significantly improved the π–π interactions with Phe274 compared with that observed for the azoxystrobin, justifying the higher potency of the compound [10].

Moreover, most of the new compounds displayed excellent in vivo fungicidal activity against *Sphaerotheca fuliginea* at the concentration of 200 μmol/L.

**Figure 5:**
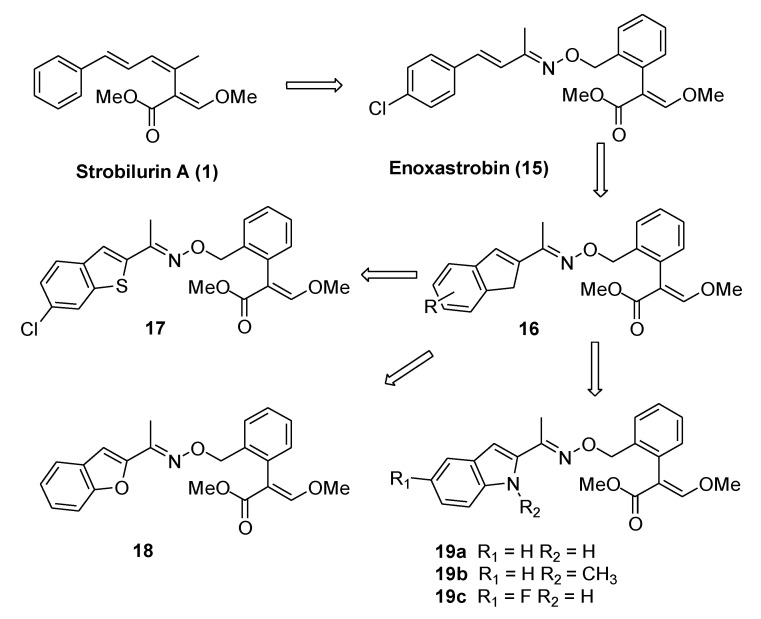
Enoxastrobin-inspired analogs.

Starting from the scaffold of enoxastrobin (**15**, Figure 5) developed by Rohm and Haas Company, Xie et al. introduced modifications in the side chain. In order to stabilize the *E*-styryl group, the authors firstly synthesized a small library of indene-substituted oxime ethers (**16**, Figure 5) [11]. Afterward, they prepared new oxime ethers featuring heterocyclic moieties, which could drive some physicochemical properties, such as lipophilicity and solubility, toward the optimal balanced range for uptake and bioavailability. In particular, benzothiophene, benzofuran, and indole analogs were developed (Figure 5) [12,13,14].

All the new synthesized compounds were tested on *Pyricularia oryzae*, *Botrytis cinerea*, *Erysiphe graminis*, *Colletotrichum lagenarium*, *Pseudoperonospora cubensis*, and *Puccinia sorghi* Schw, and some of them (e.g., **17**, **18,** and **19b**) showed fungicidal activity similar or higher than enoxastrobin (Table 1).

Various research groups synthesized novel strobilurin derivatives containing a triazole moiety. These studies focused on the introduction of 1,2,4-triazole group mainly because of its broad spectrum of biological activity [15,16,17,18,19,20]. A large number of antifungal agents containing 1,2,4-triazole group as pharmacophore inhibit the formation of the cell membrane by preventing the ergosterol biosynthesis [21].

Remarkably, the 1,2,4-triazole analogs synthesized by Chaudhary et al. and Liu et al. showed antifungal activities comparable to or better than azoxystrobin [15,17].

The authors studied the activity of 1,2,4-triazole thiol strobilurin analogs on plant pathogens (*Fusarium oxysporum*, *Magnaporthe grisea*, *Drechslera oryzae*) as well as human pathogens (*Cryptococcus neoformans* NCM3378, *Cryptococcus neoformans* NCM3542, *Aspergillus fumigatus*) [15].

**Figure 6:**
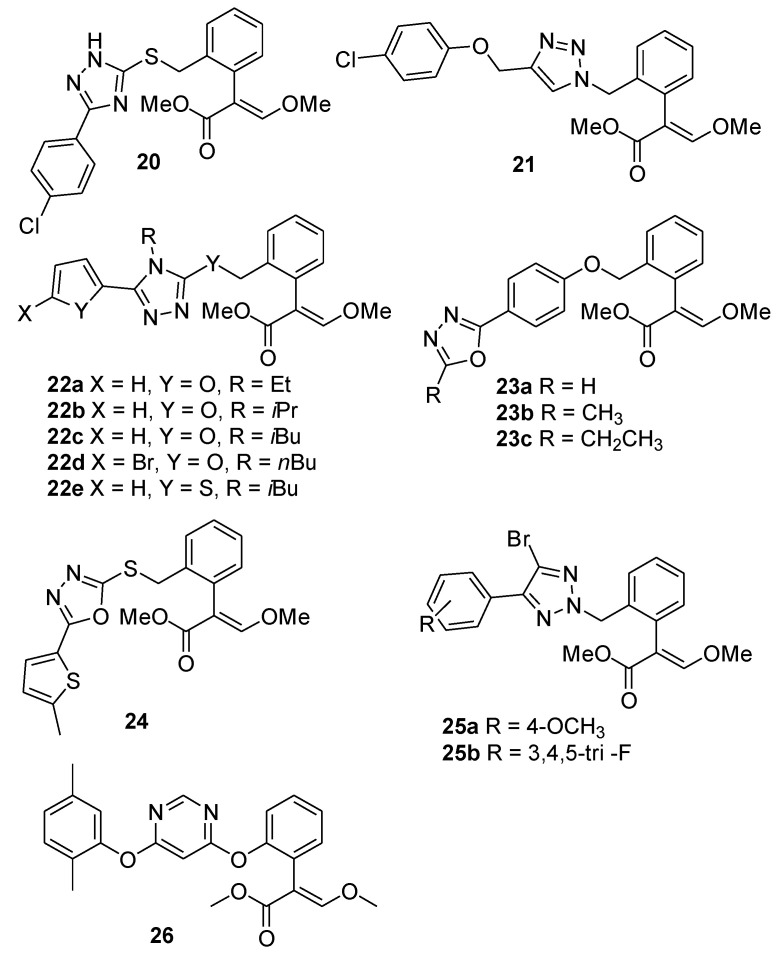
Structure of strobilurin derivatives containing a triazole moiety (**20**–**25**) and structure of azoxystrobin analog **26**.

The introduction of a chlorine atom in *para* position on the phenyl ring had a positive effect on the fungicidal activity of these compounds. The *p*-chlorophenyl derivative **20** (Figure 6) was the most effective inhibitor against all the tested pathogens with MIC (minimum inhibitory concentration) values in the range of 16-64 µg/L). The inhibition of resazurin (RZ) reduction indicated that this compound has a mechanism of action similar to azoxystrobin, inhibiting the mitochondrial respiration by binding to the Qo site of cytochrome b (EC_50_ for inhibition of RZ reduction in *D*. *oryzae* by azoxystrobin and compound **20** were 3.42 ± 0.03 µg/mL and 3.62 ± 0.21 µg/mL, respectively). At the same time, the authors observed that 1,2,3-triazole derivatives (i.e., compound **21**) were ineffective in controlling the fungal growth at the highest concentration (512 µg/mL), and concluded that the 1,2,4-triazole moiety contributed to the antifungal activity of these derivatives.

Liu and coworkers [17] obtained a small library of strobilurin 1,2,4-triazole derivatives containing furan or thiophene rings (**22a**–**e**, Figure 6), which exhibited in vitro fungicidal activities against *Physalospora piricola*, *Cercospora arachidicola* Hori, *Rhizoctonia cerealis* comparable to or higher than azoxystrobin.

Among the furanyl derivatives, compounds featuring a three- or four-carbon chain (**22b**, **22c**, Table 2) linked to the 1,2,4-triazole group had the highest inhibitory effect (Table 2). The EC_50_ values of compounds **22a** (14.82 mg/L) and **22b** (18.72 mg/L) against *Cercospora arachidicola* Hori were much lower than that of the control (40.54 mg/L). Moreover, the introduction of a bromine atom linked to the furan ring allowed to obtain compound **22d** with an inhibition rate of 90% against *Rhizoctonia cerealis*. The EC_50_ values of compounds **22d** (9.89 mg/L) and **22e** (8.66 mg/L) against *Rhizoctonia cerealis* were lower than that of azoxystrobin (10.86 mg/L).

In 2017 the same research group obtained strobilurin analogs containing the 1,3,4-oxadiazole moiety [22] (Figure 6). The antifungal activity of some representative compounds (**23a**–**c**, **24**) is reported in Table 3. In general, the efficacy of these new analogs in controlling the fungal growth of *Sclerotinia sclerotiorum* was comparable to that of azoxystrobin. Interestingly, some of them were more effective than the control against *Rhizoctonia cerealis*.

Li and coworkers designed new strobilurin analogs combining the 2-(2-methylphenyl)-3-methoxyacrylate pharmacophore with a series of 4-halo-5-aryl-1,2,3-triazoles, which had previously showed excellent fungicidal activity against *Phytophthora capsici* and *Sclerotinia sclerotiorum* [23]. The compounds exhibited moderate to good fungicidal activity against *Phytophthora capsici* and *Alternaria alternata*. In particular, compounds **25a** and **25b** (Figure 6) inhibited the fungal growth of *Phytophthora capsici* up to 73.6%, a significantly higher value than the 42.5% observed for difenoconazole.

Based on the active substructure combination approach, Liu et al. designed and synthesized new azoxystrobin analogs with various substituted phenyl groups linked to the pyrimidine ring [24]. The analogs exhibited moderate or remarkable antifungal activities against three tested fungi, *Botrytis cinerea*, *Colletotrichum orbiculare,* and *Phytophthora capsici* Leonian.

Preliminary structure–activity relationships (SAR) studies showed that the introduction of an electron-withdrawing group on the phenyl ring increased the activity against all the tested fungi with the following trend: 2-substituted phenyl derivative > 4-substituted phenyl derivative > 3-substituted phenyl derivative. Conversely, the effect of the introduction of electron-donating groups was the following (in terms of activity): 4-substituted phenyl derivative > 3-substituted phenyl derivative > 2-substituted phenyl derivative. Interestingly, among the tested compounds, the 2,5-dimethylphenyl derivative **26** (Figure 6) displayed the most promising activity, with a growth inhibition rate (%) comparable (75–78%) to that of azoxystrobin (66–71%) against *Colletotrichum orbiculare*, *Botrytis cinerea* Pers, and *Phytophthora capsici* Leonian.

**Figure 7:**
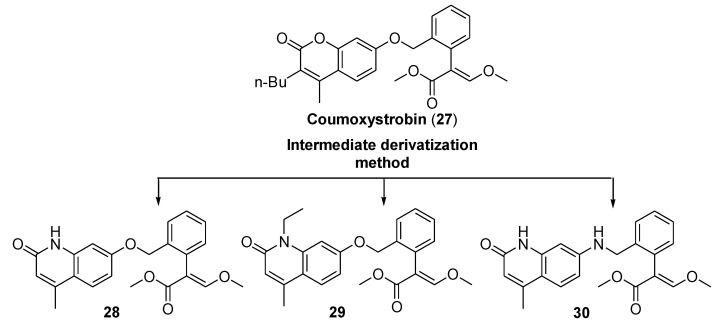
Structure of representative coumoxystrobin analogs **28**–**30** containing the quinolin-2(1H)-one moiety.

The discovery of coumoxystrobin (**27**, Figure 7) [25], containing the coumarin skeleton, paved the way to the synthesis of new strobilurins. Starting from the structure of this compound, Liu et al. investigated the activity of derivatives containing the quinolin-2(1H)-one moiety as a bioisoster of coumarin (Figure 7) and tested their efficacy in controlling the growth of ten plant pathogens [26].

The modification of the lactam ring resulted in crucial for the fungicidal activity. The introduction of the ethyl group at N1-position gave compounds more effective against all the tested fungi compared to the N-H-containing compounds (e.g., **29** vs. **28**). Compounds featuring an amino group at C7 position of the quinolinone moiety (e.g., **30**) displayed better antifungal activity against *Gibberella fujikuroi*, *Sclerotinia sclerotiorum*, *Phytophthora infestans*, *Alternaria solani,* and *Fusarium graminearum* than compounds containing an ether linkage, which, on the contrary, was beneficial to the antifungal activity against *Fusarium oxysporum* and *Rhizoctonia cerealis*.

In particular, the growth inhibition activity of compound **29** was comparable and in some cases higher than the activity of the reference coumoxystrobin. The authors reported the EC_50_ values of compounds **29** (EC_50_ 3.4646 μg/mL) and **30** (EC_50_ 8.9148 μg/mL) against *Rhizoctonia cerealis*. The results indicate that compound **29** is 4.17- and 5.07-fold more effective than coumoxystrobin (EC_50_ 14.4593 μg/mL) and azoxystrobin (EC_50_ 17.5804 μg/mL), respectively, against this pathogen.

**Figure 8:**
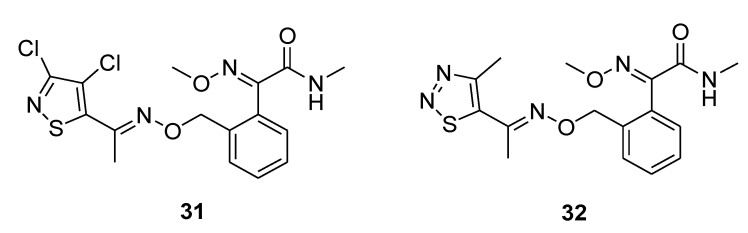
Structure of 3,4-dichloroisothiazole and 1,2,3-thiadiazole-containing strobilurins.

In 2017 Chen et al. published two papers about the synthesis of novel fungicides containing isothiazole-, thiadiazole-, and thiazole-based structures (Figure 8).

Using trifloxystrobin (**7**) structure as a template, the authors first designed and synthesized 3,4-dichloroisothiazole-containing strobilurins [27]. This heterocyclic scaffold can be found in numerous biologically active molecules [28]. In particular, the 3,4-dichloroisothiazole-5-carboxylic acid derivative isotianil was developed as a novel fungicide with activating defense responses against a wide range of plant pathogens [29].

Compound **31** (Figure 8) exhibited excellent activity with EC_50_ of 0.07 μg/mL and 0.49 μg/mL against *R*. *cerealis* and *P*. *infestans*, respectively (Table 4). These values are comparable to that of the positive control azoxystrobin. Moreover, compound **31** was more effective than azoxystrobin in controlling the growth of *G*. *zeae* and *B*. *cinerea* (Table 4).

Similarly, the same authors prepared novel 1,2,3-thiadiazole and thiazole-based strobilurins [30]. The 1,2,3-thiadiazole derivative **32** (Figure 8) exhibited excellent activities against *G*. *zeae*, *S*. *sclerotiorum*, and *R*. *cerealis* with EC_50_ values of 2.68, 0.44, and 0.01 μg/mL, respectively.

The most active compounds of each series were evaluated as fungicide candidates against *Sphaerotheca fuliginea* and *Pseudoperonospora cubensis* in cucumber fields.

Both the 3,4-dichloroisothiazole derivative **31** and the 1,2,3-thiadiazole derivative **32** showed better efficacy against cucumber *S*. *fuliginea* than azoxystrobin and trifloxystrobin at the same application rate (37.5 g ai/ha). Moreover, compound **32** showed significantly better efficacy (p < 0.05) against *P*. *cubensis* than trifloxystrobin and efficacy comparable to azoxystrobin (at the same application rate of 75 g ai/ha). Compounds **31** showed efficacy against *P*. *cubensis* comparable to pyraclostrobin but significantly better than that of trifloxystrobin at the same application rate (75 g ai/ha.).

An interesting approach to develop new strobilurin-based fungicides was recently reported by Su et al. [31]. The strategy is based on the combination of the pharmacophore moieties of strobilurins (β-methoxyacrylate, methoxyiminoacetamide, or methoxy-N-methylacrylamide) with a monoterpenic phenol typically found in plant essential oils (EOs).

Antifungal activity of EOs has been frequently associated with the presence of monoterpenic phenols, such as thymol, carvacrol, and paeonol [32]. However, EOs components are generally volatile, unstable to light or heat. Additionally, their short-term fungicidal efficacy and slow action considerably restrict their possible practical application.

**Figure 9:**
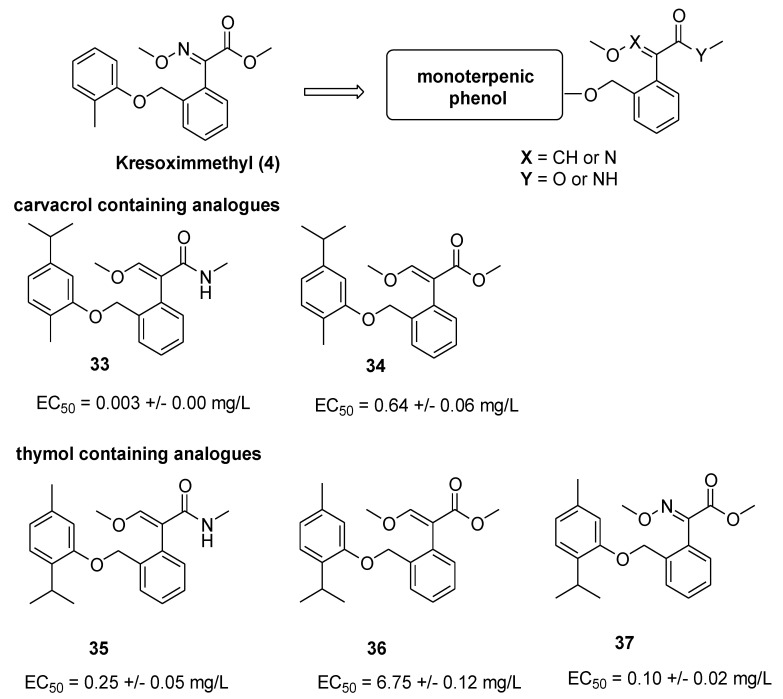
Structure of representative compounds and EC_50_ values against *Sclerotinia sclerotiourum* (azoxystrobin EC_50_ = 18.75 ± 1.10 mg/L) [EC_50_ values from Reference [31]].

The authors prepared seventeen new compounds and tested their potential fungicidal activity against eleven species of plant pathogen fungi. The structure of representative analogs containing carvacrol and thymol moiety and EC_50_ values against *Sclerotinia sclerotiourum* are reported in Figure 9. Compound **33** exhibited a very interesting activity against *Pestalotiopsis theae*, *Phomopsis adianticola*, *Sclerotinia sclerotiorum,* and *Magnapothe grisea* as well.

**Figure 10:**
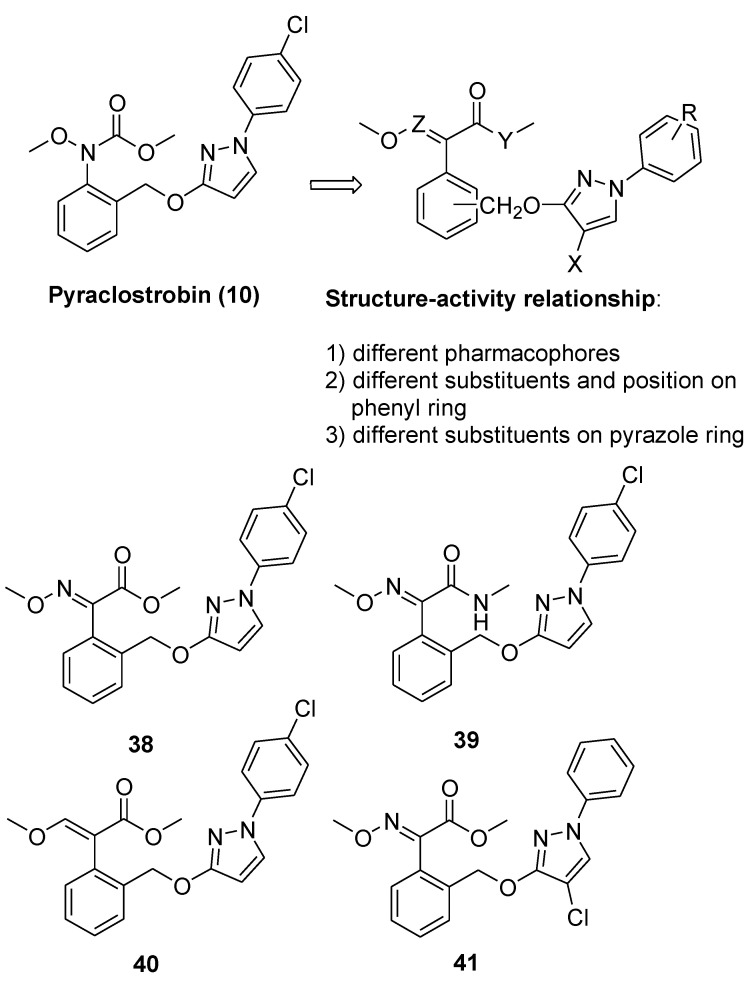
Structure of pyraclostrobin analogs from Reference [33].

Using pyraclostrobin (**10**) as a lead compound, Liu and coworkers [33] obtained 44 new strobilurin analogs by introducing the methoxyiminoacetate or the methoxyacrylate pharmacophore into halo- or (un)substituted arylpyrazole scaffolds (Figure 10). In particular, the authors focused their attention on three structural aspects: the effect of different pharmacophores and their position; the effect of nature and position of the substituent on the terminal benzene ring; the effect of halogen substituent on the pyrazole ring.

The SARs study highlighted that the combination of both methoxyiminoacetate pharmacophore and electron-withdrawing substituent R on phenyl ring improved the fungicidal activity. Moreover, the type and size of the halogen on the pyrazole ring resulted crucial for the efficacy. In particular, the presence of a chloro substituent had a positive effect on the fungicidal activity, whether it was on the pyrazole ring or on the phenyl ring. The collected biological data and the comparative molecular field analysis allowed the authors to build up a 3D-QSAR model for these new strobilurin analogs with high correlative and predictive abilities [33].

Among the obtained analogs, compounds **38**–**41** (Figure 10) exhibited 98.94%, 83.40%, 71.40%, and 65.87% inhibition rates at 0.1 μg/mL against *Rhizoctonia solani*, respectively, which are better than commercial pyraclostrobin (35.73%).

**Figure 11:**
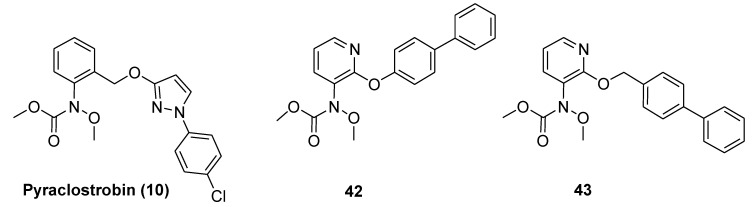
Structure of pyraclostrobin analogs from Reference [34].

In 2018, Wang et al. [34] introduced a pyridinyl group as bridge portion and side chain modifications, obtaining a library of N-*ortho* substituted pyridine analogs of pyraclostrobin (Figure 11). Besides the in vitro fungicidal activities against five important plant pathogens (*Botrytis cinerea*, *Phytophthora capsici*, *Fusarium sulphureum*, *Gloeosporium pestis,* and *Sclerotinia sclerotiorum*), the authors evaluated the percentage of inhibition, the IC_50_ values, and predicted the total binding free energy of representative compounds against porcine SCR (Table 5). Compound **43** exhibited the lowest IC_50_ value (0.95 µM). In contrast, compound **42**, without the methylene linker, displayed only 11% inhibitory activity at 100 µM concentration, suggesting that the flexible side chain is favorable for the enzymatic inhibition activity. In general, the presence of a hydrophobic side chain is critical for the antifungal activity of these analogs. Moreover, biaryl rings at the side chain are preferable and certain flexibility is beneficial to the inhibitory activity against SCR bc1 complex.

Interestingly, docking studies into the binding pocket of the cytochrome bc1 complex showed that the pyridinyl group can form stable arene-H interaction with residue proline-271, thus improving the binding to cytochrome bc1 complex.

**Figure 12:**
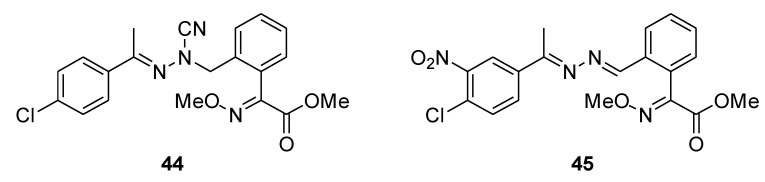
Structure of strobilurin analogs containing hydrazono-methyl moiety.

In 2016, Jia et al. investigated compounds that shared structural characteristics of strobilurin and N′-nitrohydrazinecarboximidamide, discovering compound **44** (Figure 12) as the main byproduct. The compound showed excellent fungicidal activity against *Phytophthora infestans*, *Botryosphaeria dothidea*, *Botrytis cinerea,* and several other plant pathogenic fungi. For this reason, in 2019 the same authors synthesized (E)-methyl 2-(2-((1-cyano-2-hydrocarbylidenehydrazinyl)-methyl)-phenyl)-2-(methoxyimino)acetates using **44** as a lead compound [35]. Although no new molecules resulted more active than the lead compound, they isolated compound **45** (Figure 12), which showed a broad spectrum of activity, as a byproduct of their synthetic pathway.

Recently, Matsuzaki and coworkers [36] obtained new Qo inhibitors characterized by a tetrazolinone pharmacophore and a methyl group at position 3 on the central phenyl ring. These compounds were designed to overcome the resistance developed by different pathogenic species and associated with the G143A mutation at the target site. The presence of the methyl group of the alanine residue at position 143 seems to cause severe steric hindrance at the central linking rings of QoIs.

**Figure 13:**
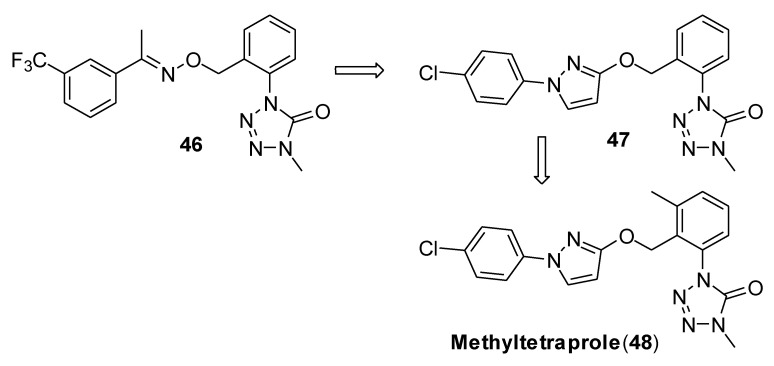
Structure of strobilurins analogs containing the tetrazolinone pharmacophore (**46**–**48**).

**Figure 14:**
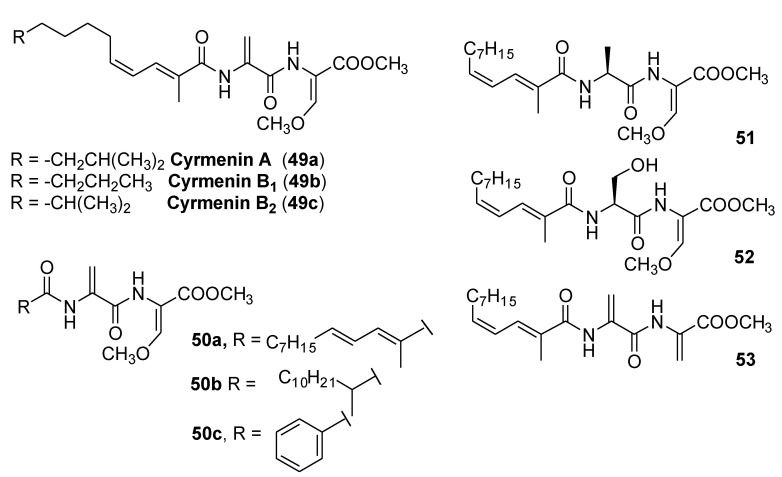
Structure of natural cyrmenins and analogs.

Through an initial random screening on 200 QoI-like compounds to identify the key structural moieties to retain the activity against G143A mutant, the authors found that compound **46** (Figure 13), featuring the tetrazolinone pharmacophore and the same side chain of trifloxystrobin, was less affected by this mutation, showing a resistant factor RF = 2 (Figure 13, Table 6). After a careful structure–activity relationship investigation, the authors obtained compound **47** (Figure 14) with the same side chain of pyraclostrobin (4-chlorophenylpyrazole structure). The EC_50_ of compound **47** in the wild-type sensitive strain (WT) and G143A mutant were both 0.02 ppm, showing a 10- to 20-fold increase of activity compared to compound **46** (Table 6).

Finally, the introduction of the 3-methyl group on the central phenyl ring led to obtain methyltetraprole **48**, showing a 10-fold higher activity than that of compound **47** (Table 6). The authors hypothesized that the presence of the methyl group might have a role in limiting the rotation of the side chain and in avoiding steric hindrance between the QoI and the mutated target site.

## 3. Other Natural Products Inhibiting the Cytochrome bc1 Complex

### 3.1. Cyrmenin A, B_1_ and B_2_

Cyrmenins A, B_1,_ and B_2_ (**49a**–**c**, Figure 14) are antifungal metabolites isolated from the culture broth of myxobacteria *Cystobacter armeniaca* and *Archangium gephyra*. These modified N-acyldipeptide compounds contain a dehydroalanine, a 2-amino-3-methoxyacrylate moiety, and a (2*E*, 4*Z*)-undecadienoic or dodecadienoic acid residue. Cyrmenines exhibit high antifungal activity, showing at the same time exceptionally low toxicity for animal cell cultures [37].

The first total synthesis of cyrmenin B_1_ (Figure 14) was reported in 2009 [38] and the same authors reported the synthesis of some representative derivatives for structure–activity relationship studies [39]. The antifungal activity of cyrmenin B_1_ and analogs (**50**–**53**) was tested against *Saccharomyces cerevisiae* Meyen ex Ec. Hansen, strain IPV 637, *Aspergillus niger* Tegh., strain IPV F303, *Botrytis cinerea* Pers., strain IPV F5.2, *Cochliobolus miyabeanus*, (S.Ito and Kurib) Drechsler ex Dastur, and *Pyricularia oryzae* Cavara, strain IPV A1.

The bioassay results clearly indicated the relevance of each portion of the parent molecule (lipophilic unsaturated chain, dehydroalanine moiety, and β-methoxyacrylate system), leaving very little space for synthetic modifications.

Interestingly, only the modification of the conjugated double bond geometry (cyrmenin B_1_ vs. its (8*E*,10*E*)-geometrical isomer **50a**) was tolerated. The complete lack of activity of compounds **50b** and **50c** underlined that maintaining the lipophilicity of the molecule was not enough to have active compounds. The exo double bond of the parent molecule was also crucial for the antifungal activity. In fact, both compound **51**, bearing an alanine residue, and the compound bearing a serine residue (**52**) were inactive against all the tested strains. The modification of the expected crucial β-methoxyacrylate group was also deleterious (compound **53**). All these results evidenced a limitation for the development of new fungicide compounds derived from natural cyrmenins.

### 3.2. Mixothiazols, Melithiazols and Fulvuthiacens

Myxobacteria are a rich source of various biologically active secondary metabolites. These include the antifungal agents myxothiazol and melithiazol, which are very potent inhibitors of the electron transport through the cytochrome bc1 complex of the eukaryotic respiratory chain.

**Figure 15:**
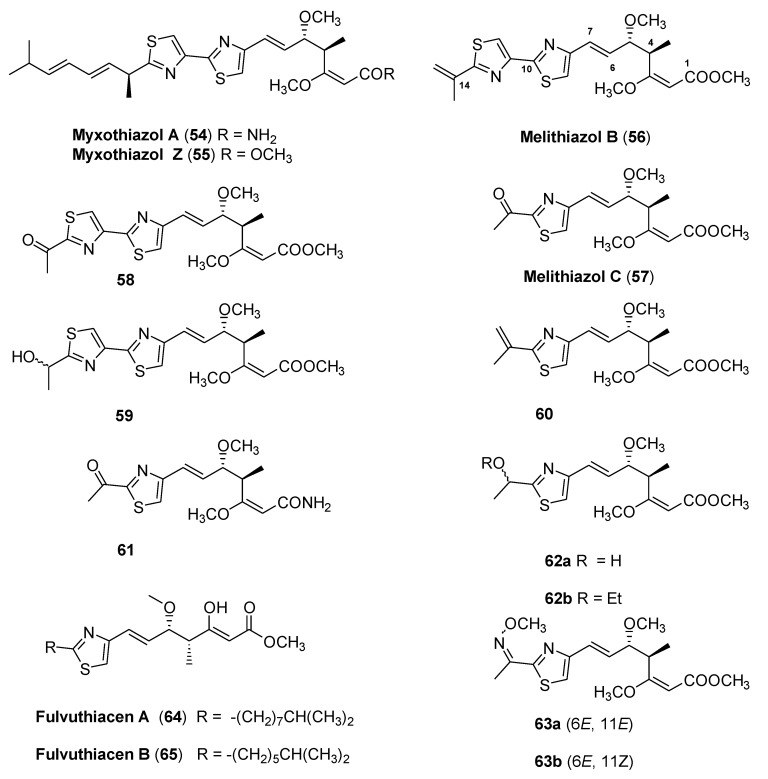
Structures of mixothiazols, melithiazols, and fulvuthiacens.

In 1980, Reichenbach et al. and Hofle et al. reported the isolation of a novel myxobacterial antibiotic named myxothiazol A (**54**) [40,41], whose structure is characterized by a central bis-thiazole unit linked to a β-methoxyacrylate moiety and a heptadienyl side-chain bearing a stereogenic center at the α-position of the thiazole ring (Figure 15). Acting as potent inhibitors of the electron transport through the cytochrome bc1 complex, both myxothiazol A (**54**) and its corresponding methyl ester, myxothiazol Z (**55**), exhibit broad antifungal activity as well as significant cytotoxicity against different human tumor cell lines with IC_50_ values reaching as low as 0.01 ng/mL [42,43]. The high mammalian toxicity hampered the development of myxothiazol synthetic derivatives. During the following three decades, more than 20 structurally related fungicides have been isolated from various strains of myxobacteria, including the congeneric melithiazols.

Melithiazols A–N contain a β-substituted β-methoxyacrylate pharmacophore and act as inhibitors of the cytochrome bc1 complex as well [44].

Unlike myxothiazols, all melithiazols occur only as methyl esters and lack the lipophilic heptadienyl side chain. Moreover, the greatly reduced mammalian toxicity of these metabolites with respect to the toxicity of myxothiazols makes melithiazols interesting for the development of new fungicides. Unfortunately, due to the poor amounts of melithiazols usually obtained from fermentation, it has not been possible to develop a derivatization program to investigate structure–activity relationships. However, synthetic efforts to obtain melithiazol B (**56**) [45] and C (**57**) [46] through oxidative degradation of myxothiazol A and reductive cleavage of a thiazole ring, respectively, allowed to test the biological activities of intermediates and derivatives, to obtain some structure–activity correlations.

Concerning melithiazol B (**56**) and its derivatives, the authors highlighted that compounds with an ester pharmacophore and a short polar side-chain (e.g., compounds **58** and **59**) showed high antifungal activity (Table 7). Moreover, in general, the cytotoxicity decreased significantly by decreasing the lipophilicity of these molecules.

Structure–activity relationship studies on melithiazol C (**57**) derivatives showed that the amide analog (**61**) and the 14-hydroxy derivative lost the antifungal activity (**62a**), whereas the 14-ethoxy derivative (**62b**) showed good antifungal activity and only slightly increased cytotoxicity. Moreover, the (6*Z*)-isomer of melithiazol C was essentially inactive in all test systems.

The vinyl derivative **60** and the (11*E*) and (11*Z*) methoximes **63a** and **63b** showed an exceptionally high antifungal activity and low cytotoxicity, comparable to that of the commercial fungicide kresoxim-methyl. 

Recently, Panter and coworkers [47] elucidated the structure of two new secondary metabolites (fulvuthiacene A (**64**) and B (**65**), Figure 15) isolated from *Myxococcus fulvus* MCy9280 by combining the statistics-based mining of mass spectrometry approach and multidimensional NMR spectroscopy.

These compounds contain a terminal β-methoxymethyl acrylate moiety and their evaluation provided new insights into the overall structure–activity relationship picture of the β- methoxyacrylate class of bc1 complex inhibitors.

Indeed, fulvuthiacene A and B showed very poor activity against a panel of fungi and yeast. Comparing the NADH oxidation IC_50_ values of known naturally occurring methoxymethacrylate type respiratory chain inhibitors, the authors observed that a wide variety of residues are tolerated in C-2 position of the β-methoxymethacrylate pharmacophore (strobilurin A IC_50_ = 83 ng/mL, oudemansin IC_50_ = 400 ng/mL, and cyrmenin A IC_50_ = 27 ng/mL). On the contrary, for compounds bearing a C-3 extended β-methoxyacrylate warhead, the structural requirements are very narrow and a bisthiazol or thiazolinethiazol moiety is needed to produce a strong bc-1 inhibitor (melithiazol B IC_50_ = 18 ng/mL, melithiazol C IC_50_ = 1600 ng/mL, fulvuthiacene A no inhibition of NADH oxidation up to 64,000 ng/mL). The results reported in this study might thus serve as starting points for the development of the next generation of β-methoxymethacrylate fungicides.

### 3.3. Miuraenamides

**Figure 16:**
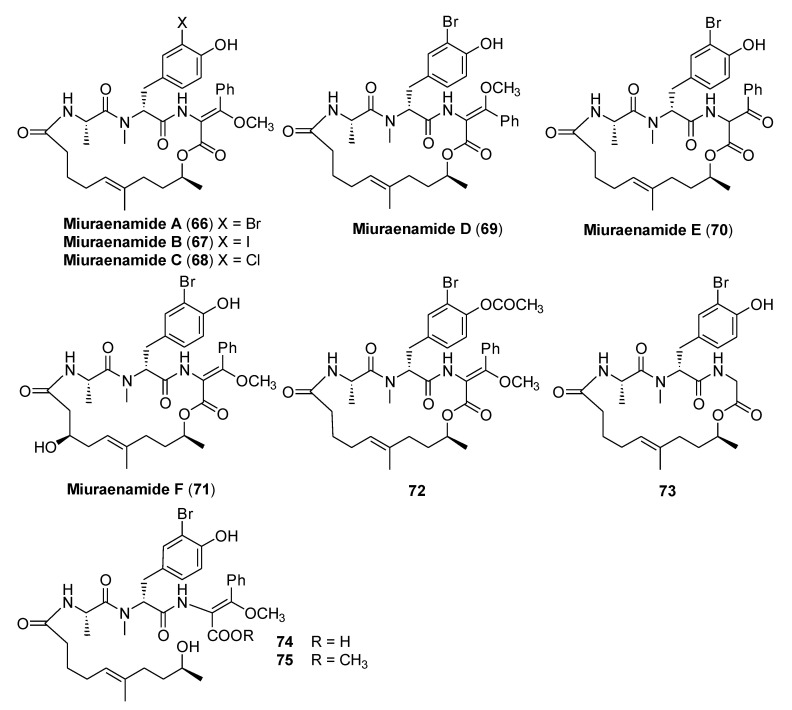
Structures of miuraenamides A-F (**66**–**71**) and related derivatives (**72**–**75**).

Miuraenamide A–F (**66**–**71**, Figure 16) are cyclic hybrid polyketide-peptide antibiotics isolated from *Paraliomixa miuraensis*, a slightly halophilic myxobacterium discovered by Ojika et al. at the seashore on Miura Peninsula in Kanagawa, Japan [48,49]. The biological properties of these natural products included antimicrobial activity and inhibitory activity against NADH oxidase.

Structure–activity relationship studies on miuraenamides A–F and related derivatives were conducted on the phytopathogen *Phytophthora capsici* (Table 8). The authors highlighted that the type of halogen present in the tyrosine residue in miuraenamides A–C is not important for their activity. Moreover, the presence and the geometry of the β-methoxyacrylate moiety (C12–C14, compounds **69**, **70,** and **74**) affects the activity of these compounds. The lipophilicity of the polyketide moiety and the free phenol group on the peptide portion of the structure seem to be important for the activity of these compounds, as demonstrated by the low activity observed for miuraenamide F (**71**) and acetate **72**. The macrocyclic structure of the miuraenamides is essential as well, with the anti-*Phytophthora* activity being completely lost in the case of the ring-opened derivatives **74** and **75**, although these compounds contain the β-methoxyacrylate moiety.

### 3.4. Crocacins

**Figure 17:**
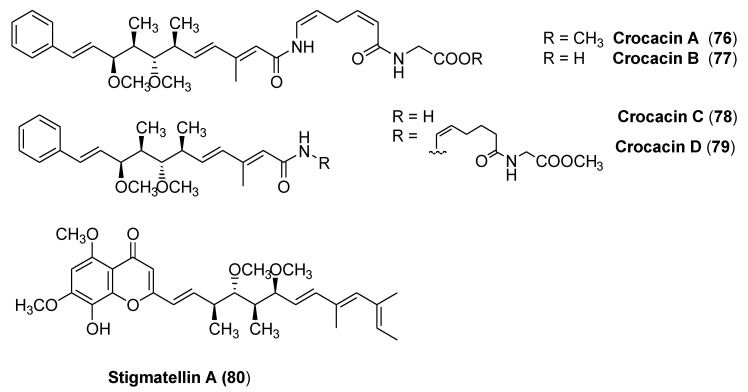
Structures of crocacins A–D (**76**–**79**) and stigmatellin A (**80**).

In 1994, crocacins A, B, C, and D (**76**–**79**, Figure 17) were isolated from the myxobacterium *Chondromyces crocatus*. The compounds crocacin A and D inhibited the electron transport chain at complex III in a beef heart mitochondrial respiration assay and inhibited the growth of several fungi in vitro. [50]. Although natural crocacins had interesting biological activity, their practical use would be severely limited by their physical properties, such as the very poor photostability and their structural complexity. However, the emergence of resistance to different pathogenic species associated with G143A mutation renewed the interest toward crocacin A, as it showed little or no cross-resistance to a strobilurin-resistant strain of *Plasmopara viticola* in small vine plants in the glasshouse, or against a strain of yeast engineered with the G143A mutation.

Crowley and coworkers [51] studied the binding of crocacins to the active site in order to design structurally simpler and more stable synthetic analogs. An inhibitor binding model to the mammalian cytochrome bc1 complex was constructed, which was consistent with X-ray crystallographic analysis of an analog bound to the chicken heart cytochrome bc1 complex. The proposed binding mode for the crocacins combined some binding features of stigmatellin A (**80**, Figure 17, a potent inhibitor of the quinol oxidation (Qo) site of the cytochrome bc1 complex) and some binding features of strobilurins. The differences in binding between the crocacins and methoxyacrylate stilbene could explain the apparent lack of cross-resistance of the crocacins with strobilurins.

The binding site model, coupled with further molecular modeling, was used to design analogs of crocacins A (**76**) and D (**79**), with mixed results. To improve the stability of the synthetic analogs, efforts were made to replace the three double bonds, which most likely could give rise to significant instability to sunlight, with more robust groups, such as aromatic rings. This strategy had been successful in the development of stable analogs of the strobilurins.

Replacement of the side chain with simple n-alkyl chains or with side chains containing aromatic rings (e.g., *n*-alkoxybenzamide) gave compounds very active in the beef heart mitochondrial respiration assay but inactive as fungicides. Further modeling showed that substituting the benzamide with a 4-substituted benzyl group gave a very good overlay with the crocacin side chain. Some compounds (**84**–**87**) showed not only potent inhibition of respiration but also activity on vine downy mildew on small vine plants similar to or better than crocacins (Table 9).

The fact that some of the designed compounds were inactive despite good modeling into the active site highlights the limitations of a rather simple model based on shape fit, the complementarity of hydrogen bonding groups, and ligand conformational analysis, as the authors themselves suggest. Actually, other important factors, such as solvation and entropic effects, could be critical in binding interactions.

Nevertheless, the design strategy was successful in that analogs of the naturally occurring fungicide crocacin D (**79**) were synthesized. These compounds were active both in a respiration assay, on fungi, and on plants, and were significantly more stable than the natural compounds.

### 3.5. Neopeltolide

**Figure 18:**
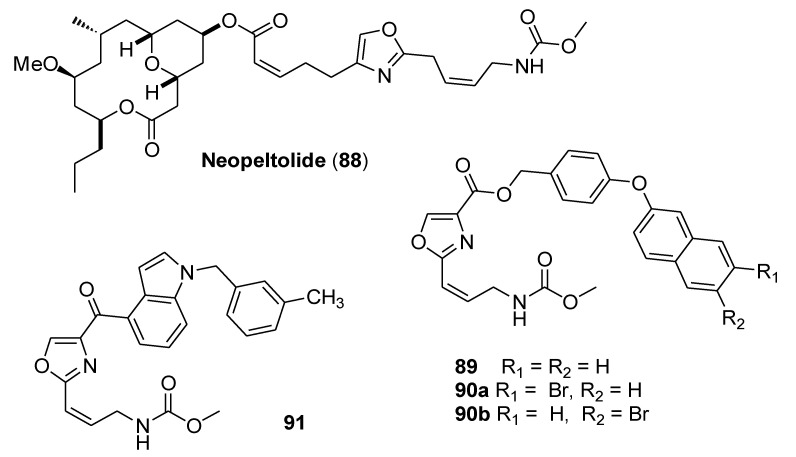
Structure of neopeltolide (88) and representative simplified analogs (**89**, **90a**,**b** and **91**).

Neopeltolide (**88**, Figure 18), a marine natural product isolated in 2007, was found to inhibit the bovine heart mitochondrial cytochrome bc1 complex with an IC_50_ value of 2.0 nM [52]. However, its complex structure prevented the obtainment of the compound by chemical synthesis. SAR studies showed that the carbamate-containing oxazole moiety was the key structural feature, whereas the 14-membered macrolactone moiety did not make a significant contribution to binding.

Zhu and coworkers [53] determined the binding mode of neopeltolide by integrating molecular docking, molecular dynamics (MD) simulations, and molecular mechanics Poisson-Boltzmann surface area calculations (MM/PBSA), which showed that neopeltolide is a Qo site inhibitor of the bc1 complex. Based on neopeltolide binding mode, structural modification was carried out with the aim to simplify its structure. Thus, a series of new neopeltolide derivatives with much simpler chemical structures were rationally designed and synthesized.

Compound **89** bearing a naphthylether fragment in place of the structurally complex 14-membered macrolactone moiety showed high inhibitory activity against porcine SCR, with an IC_50_ value of 0.047 μM. The introduction of a bromo group on the naphthalene ring (compounds **90a** and **90b**) further improved the activity against SCR. Compound **90a** was the most potent candidate against SCR (IC_50_ = 12 nM, Table 10) [53].

Molecular docking of the newly synthesized compounds was performed, followed by MD simulations and MM/PBSA calculations. Computational simulations revealed that most of the compounds inside the Qo site of the bc1 complex formed a hydrogen bond with Glu271 and a π-π interaction with Phe274, while the most active compound (**90a**) formed an additional hydrogen bond with His161.

Recently, the same authors prepared a new set of analogs by replacing the 14-membered macrolactone ring with an indole ring linked to the carbamate moiety by an ester or an amide bond [54]. The new analogs exhibited IC_50_ values ranging from 0.70 to 1.75 µM, and compound **91** showed the highest inhibitory activity against porcine SCR (IC_50_ 0.70 µM).

### 3.6. Chromanols

In the search for non-redox effects of chromanols (vitamin E-related compounds), it was noted that chromanols share structural similarity to stigmatellin (**80**, Figure 17), e.g., the chroman core.

**Figure 19:**
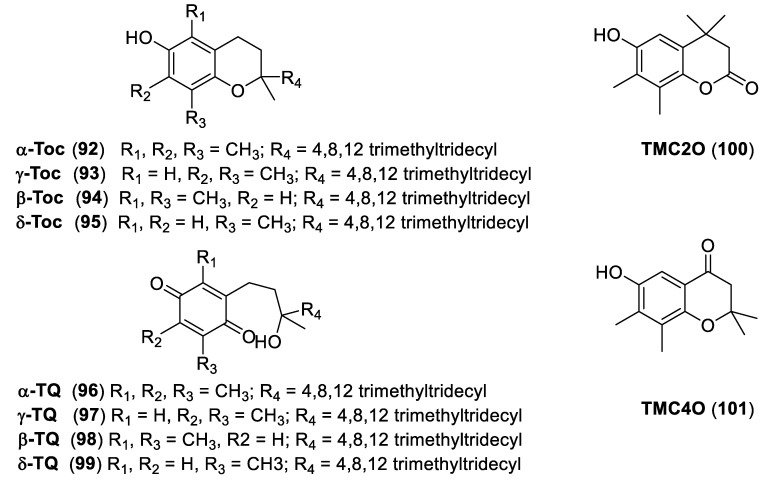
Structures of natural tocopherols (α, β, γ-, and δ-Toc, **92**–**95**), of tocopheryl quinones (α, β, γ -, and δ-TQ, **96**–**99**), and low molecular weight analogs (**100** and **101**).

Based on this common structural feature, Mullebner and coworkers [55] studied the extent and the mechanism of inhibitory effects of natural tocopherols (α, β, γ-, and δ-Toc, **92**–**95**), their oxidation products tocopheryl quinones (α, β, γ-, and δ-TQ, **96**–**99**), and synthetic (low molecular weight TQ analogs and chromanones) compounds **100** and **101** (Figure 19).

The authors found by enzymatic experiments that the 6-hydroxychroman structures of Toc-related compounds and the *para*-benzoquinone structure of TQ-related molecules can modulate the function of the mitochondrial cyt bc1 complex.

For both Toc and TQ, an incomplete methyl substitution (β, γ-, and δ-congeners) increased the inhibitory potential. This affinity was further enhanced by the introduction of keto groups into the chroman ring, thus leading to chromanones. For TMC2O (6-hydroxy-4,4,7,8-tetramethylchroman- 2-one, **100**), it was shown that it binds to the Qo pocket of the cyt bc1 complex and delays the electron transfer from dUQH2 to cyt c1 in the complex.

Docking experiments were performed to study the interaction of **100** with the cyt bc1 complex, which was similar but not identical to that of stigmatellin. 

### 3.7. Karrikinolide

Karrikinolide (3-methyl-2H-furo[2,3-c]-pyran-2-one, KAR1, **102**) is a natural butenolide found in the smoke of burning plant material, which promotes the seed germination of a wide range of plants [56]. The molecular simplicity, structural stability, and the novel skeleton of **102** inspired the synthesis of numerous analogs in order to study their efficacy in promoting seed germination [57].

**Figure 20:**
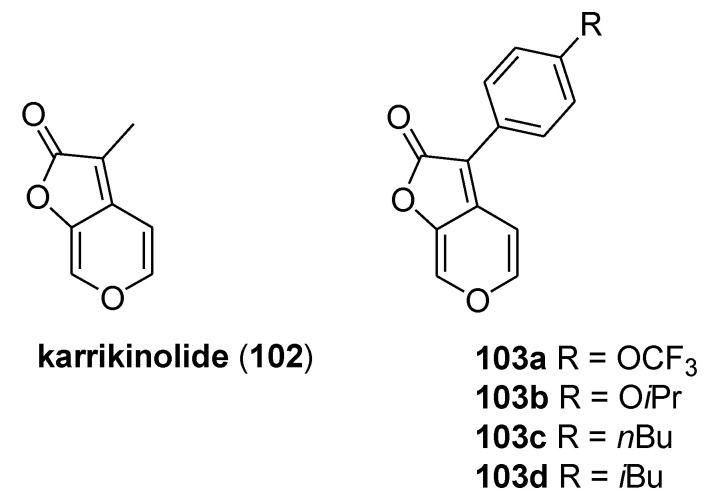
Karrikinolide (**102**) and selected derivatives **103a**–**d** from Reference [59].

In 2016, Chen et al. exploited this scaffold with the aim to develop new cytochrome bc1 complex inhibitors and reported the synthesis of 20 karrikinolide derivatives by introducing different functional groups at C3-position (Figure 20) [58]. The inhibitory activity of the newly prepared compounds was tested against SCR. SCR is composed of respiratory complex II (SQR) and complex III (the bc1 complex), which are believed to form a complex II–complex III supercomplex. Out of the tested derivatives, compound **103a**–**d** exhibited limited activity against SQR at a concentration of 10 mM (Table 11). On the other hand, their inhibitory activities against SCR and the bc1 complex support the hypothesis that these compounds act as inhibitors of the bc1 complex. In spite of the promising results, further studies will be necessary to confirm the antifungal activity of the compounds.

### 3.8. Picolinamide UK-2A

**Figure 21:**
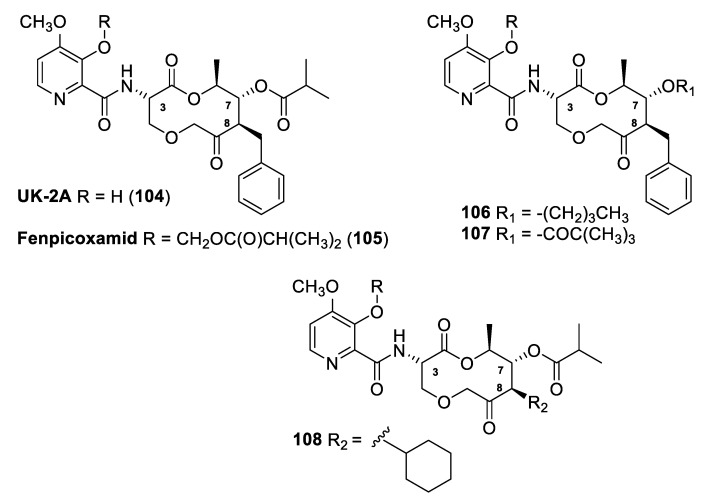
Structure of natural picolinamide UK-2A (**104**), of fenpicoxamid (**105**), and structures of representative analogs (**106**–**108**).

The natural compound picolinamide UK-2A (**104**, Figure 21) is a pyridine carboxamide originally isolated from fermentation broths of the actinomycete *Streptomyces* sp. 517–02 [59]. Pyridine carboxamides inhibit mitochondrial respiration by binding to the Qi ubiquinone site of the cytochrome bc1 complex. Remarkably, no target site-based cross-resistance with Qo inhibitors was observed for these compounds. However, to date only two molecules acting as Qi site inhibitors have been commercialized: cyazofamid and amisulbrom as oomycete-specific substances.

Picolinamide UK-2A was used as a lead compound to design new macrocyclic fungicidal molecules, and recently at Dow AgroSciences LLC the novel fungicide fenpicoxamid (**105**, Inatreq™, Figure 21) was developed by semi-synthetic modification of the natural compound.

The acyloxymethyl ether derivative fenpicoxamid showed a broad spectrum of activity in in vitro assays and excellent efficacy on *Zymoseptoria tritici* (synonym, *Mycosphaerella graminicola*, wheat leaf blotch), the pathogen of greatest concern for wheat production in Europe.

Recently, Owen and coworkers [60,61,62] described structure–activity relationship (SAR) studies on UK-2A, analyzing the impact of modifications of the macrocycle isobutyryl ester position, ring replacement, and modifications to the macrocycle benzyl position. Moreover, the relative activities of the new analogs were rationalized, based on a homology model constructed for the *Z*. *tritici* Qi binding site.

In particular, the isobutyryl ester of UK-2A was replaced by a series of ester groups and a set of ether groups, carbonate, and carbamate moieties. The authors reported that linkages other than esters are well tolerated at the 7-position of the macrocycle, with the only exception of the carbamate analogs. The *n*-butyl ether (**106**) was the most active analog evaluated, and compound **107** (pivaloate ester) was the most active among the ester derivatives (Table 12).

The 3-hydroxy-4-methoxypicolinic acid moiety of UK-2A can be replaced by a variety of *o*-hydroxy-substituted arylcarboxylic acids, while retaining strong activity against *Z*. *tritici* and other relevant fungi.

Among the analogs featuring modifications to the macrocycle benzyl position, the cyclohexyl analog (**108**) was the most active derivative on mitochondrial electron transport (MET) assays (IC_50_ 1.23 nM) and inhibited the in vitro growth of *Zymoseptoria tritici* (EC_50_ 2.8 ppb) and *Leptosphaeria nodorum* (EC_50_ 6.2 ppb) more strongly than UK-2A (EC_50_ 5.3 and 11.3 ppb for *Z*. *tritici* and *L*. *nodorum*, respectively). On the contrary, the introduction of heterocyclic ring systems and polar linker functionalities on this position resulted in substantial loss of activity.

## 4. Conclusions

There is an urgent need for new compounds that specifically target pathogenic fungi due to the increasing rates of resistance to the drugs currently available on the market. The cytochrome bc1 complex is one of the most important fungicidal targets. Inhibitors of the cytochrome bc1 complex have been broadly studied, in particular for controlling fungal diseases. In this context, natural products have played a crucial role. Commercial analogs of the natural compound strobilurin are among the most successful classes of agricultural fungicides, with a dominant position on the global market.

This review provides an outline of advances in the investigation of new bc1 complex inhibitors based on natural products. Emphasis has been given to studies involved in the optimization of the natural scaffold. In fact, despite their enormous potential, natural compounds are often characterized by structural complexity, toxicity, and unfavorable bioavailability, which can limit their investigation and severely hamper their development. For this reason, this review focuses on those modifications of the natural core that have expanded the potency and stability of analogs compared to the parent compounds, opening the possibility of further development.

In this context, it should be stressed that, besides the in vitro antifungal test (percentage of inhibition of fungal growth or EC_50_ values), a significant number of enzymatic tests to evaluate the cyt bc1 inhibitory activity were performed using as a model system the porcine succinate cytochrome c reductase, a mixture of respiratory complex II, and bc1 complex [10,34,50,52,53,54,56]. Despite the general lack of data around the fungal cytochrome bc1 complex, those studies were also included in the review in order to obtain a complete overview of the SAR investigations on cytochrome bc1 complex inhibitors.

The history of strobilurins clearly demonstrates the value of a detailed structure–activity investigation in the context of a strategy focused on the variation of molecular structures to optimize the biological profile of the parent molecule. From the analysis of all the reported studies, it has emerged that the knowledge of the target binding site is often crucial to define the effect of substituent changes and predict modifications for enhanced potency, safety, and circumvention of resistance.

However, a major concern in the development of new cytochrome bc1 complex inhibitors is related to the toxicity to non-target organisms., e.g., plants and animals. In some cases, the evolutionary conservation of sequence and structure of the key functional subunits of the respiratory chain complexes is reflected in a low selectivity of electron transport inhibitors in different species [6]. In fact, it has recently been demonstrated that even strobilurins, which are allegedly non-toxic to humans, can also exert their mode of action in mammalian cells [63].

Despite the improved understanding of the existing targets and their inhibitors given by crystallographic and structural biology studies [64], the design of novel inhibitors of the bc1 complex with appropriate species selectivity still remains a very challenging goal. A few papers report efforts towards this aim. Monzote et al. [65] and Rotsaert et al. [66], found a certain degree of selectivity in the inhibition of the cyt bc1 activity of different species by selected molecules.

Nevertheless, it should be stressed that the relevance of the molecular target of a fungicide may change during the life cycle of the treated organism. As a consequence, different development stages may exhibit different sensitivities to a given molecule. The ATP energy-demanding spore germination is, compared to mycelial growth, particularly sensitive to respiration inhibitors such as strobilurins. Since, among eukaryotes, spore germination occurs almost exclusively in fungi, this also contributes, on the physiological level, to selective action against fungal pathogens [6].

It should be also considered that careful optimization of the biological profile of the antifungal compounds—including modulation of their pharmacodynamic and pharmacokinetic properties—can contribute to the reduction of mammalian toxicity.

Overall, recently reported results highlight the importance of cytochrome bc1 complex as a fungicidal target and spark a renewed interest in natural products as sources for new antifungal molecules. However, a great effort to increase species selectivity and to reduce the toxicity of fungicides is still needed and remains of primary importance for future antifungal drug discoveries.

## Figures and Tables

**Table 1:** In vitro and in vivo fungicidal activity (inhibition %) of representative indene (**16a**), benzothiophene (**17**), benzofuran (**18**), and indole (**19a**–**c**) -substituted oxime ethers. from References [11,12,13,14].

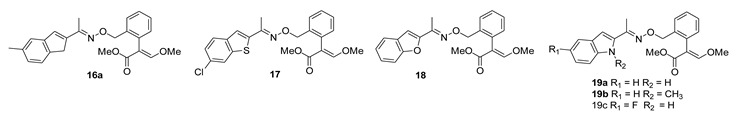					
	In vitro		In vivo		
	25 mg/L		6.25 mg/L		
**Cpd**	*Pyricularia orizae*	*Botrytis cinerea*	*Erysiphe graminis*	*Colletotrichum lagenarium*	*Puccinia sorghi* Schw.
**16a**	100	50	100	70	/
**19a**	80	80	50	0	100
**19b**	100	80	95	80	90
**19c**	100	80	99	75	60
enoxastrobin	50	100	100	85	100
	In vitro		In vivo		
	6.25 mg/L		6.25 mg/L		
**Cpd**	*Pyricularia orizae*	*Botrytis cinerea*	*Erysiphe graminis*	*Colletotrichum lagenarium*	*Puccinia sorghi* Schw.
**17**	50	100	50	100	70
**18**	/	100	100	98	40
enoxastrobin	50	100	100	90	98

**Table 2:** Fungicidal activity (growth inhibition rate, %) of compounds **22a**–**e** at 50 mg/L in vitro from Reference [17].

Compd	X	Y	R	*PP ^a^*	*CH ^a^*	*RC ^a^*	*CO ^a^*	*SS ^a^*	*FG ^a^*
**22a**	H	O	C_2_H_5_	60.0	87.0	80.3	38.9	76.3	54.8
**22b**	H	O	*i*-C_3_H_7_	60.0	84.8	83.1	36.1	80.3	42.9
**22c**	H	O	*i*-C_4_H_9_	43.3	73.9	87.3	19.4	31.6	38.1
**22d**	Br	O	*n*-C_4_H_9_	63.3	69.6	90.1	25.0	82.9	61.9
**22e**	H	S	*i*-C_4_H_9_	56.7	50.0	94.4	33.3	56.6	57.1
**AZ**				63.3	56.5	81.7	72.2	82.9	73.8

^a^ PP: *Physalospora piricola*; CH: *Cercospora arachidicola* Hori; RC: *Rhizoctonia cerealis*; CO: *Colletotrichum orbiculare*; SS: *Sclerotinia sclerotiorum*; FG: *Fusarium graminearum*; AZ: azoxystrobin.

**Table 3:** Inhibition of fungal growth (%) of compounds **23a**-**c** and **24** at 50 mg/L and EC_50_ (mg/L) against *Sclerotinia sclerotiorum* and *Rhizoctonia cerealis* from Reference [22].

	% Inhibition (50 mg/L)		EC_50_ (mg/L)	
Compd	*Sclerotinia sclerotiorum*	*Rhizoctonia cerealis*	*Sclerotinia sclerotiorum*	*Rhizoctonia cerealis*
**23a**	100.0	98.8	7.67	15.93
**23b**	98.8	97.7	7.35	9.35
**23c**	100.0	87.2	6.15	13.45
**24**	82.7	95.3	/	9.20
azoxystrobin	96.3	70.9	4.67	22.86

**Table 4:** EC_50_ value of compounds **31** and **32** from References [27,30].

	EC_50_ (µg/mL)				
	*R.c* ^a^	*P.i* ^a^	*G.z* ^a^	*B.c* ^a^	*S.s* ^a^
**31**	0.07	0.49	1.75	0.15	/
**32**	0.01	/	2.68	/	/
AZ	0.06	0.040	6.92	6.31	4.04

^a^ R. c, *Rhizoctonia cerealis*; P. i, *Phytophthora infestans* (Mont) de Bary; G. z, *Gibberella zeae*; B. c, *Botrytis cinerea*; S. s, *Sclerotinia sclerotiorum*; AZ, azoxystrobin.

**Table 5:** Percentage of inhibition, IC_50_ values, and predicted total binding free energy of selected compounds against porcine SCR from Reference [34].

Compd	% Inhibition (100 µM)	IC_50_ ± SD (µM) ^a^	ΔG_pred._^b^ (Kcal/mol)
**42**	11	/	−23.84
**43**	99.9	0.95 ± 0.012	−44.17
pyraclostrobin	99.9	1.76 ± 0.17nM	−35.85
penthiopyrad	95	1.56 ± 0.12	

^a^ Values are the mean ± standard deviation (SD) of three replicates. ^b^ Predicted binding free energies between each compound and cytochrome bc1 complex (PDB ID: 3TGU, ein = 1.0).

**Table 6:** In vitro antifungal tests against sensitive wild-type and G143A mutant of *Zymoseptoria tritici* from Reference [36].

Cpd	Sensitive Wild Type EC_50_ (ppm) ^a^	G143A Mutant EC_50_ (ppm)	Resistant Factor ^b^
strobilurin A	0.02	0.2	10
pyraclostrobin	0.001	0.2	200
**46**	0.2	0.4	2.0
**47**	0.02	0.02	1.0
methyltetraprole (**48**)	0.002	0.002	1.0

^a^ The values represent the 50% effective concentration (EC_50_) determined from two biological replicates. The 95% confidence intervals of all EC_50_ values ranged between 50% (MIN) and 200% (MAX) of the representative values. ^b^ Resistance Factor: ([EC_50_ of resistant strain]/[EC_50_ of sensitive strain]).

**Table 7:** Biological activities and lipophilicity of selected myxothiazol and melithiazol derivatives. ^[a]^.

Cpd	*Botrytis cinerea* Inibition Zone at 2 µg/disc (mm)	Citotoxicity IC_50_ (ng/mL) ^[b]^	Inibition of NADH Oxidation IC_50_ (ng/mL) ^[c]^	Lipophilicity Log P_OW_^[d]^
**54** ^[e]^	16	1	11	5.29
**55**	13	2	17	7.17
**56**	29	20	18	4.75
**58**	39	55	29	3.37
**59**	19	220	37	2.36
**57**	18	700	730	2.92
**60**	35	2000	250	3.97
**63a**	42	500	42	3.62
**63b**	42	400	85	3.81
Kresoxim-methyl ^[e]^	33	400	72	3.70

^[a]^ from References [45,46]. ^[b]^ The cytotoxicity was measured by an MTT assay with the mouse fibroblast cell line L929. ^[c]^ The inhibition of the NADH oxidation was measured with submitochondrial particles isolated from beef heart. ^[d]^ Estimated by RP-18 TLC. ^[e]^ Values taken from Reference [44].

**Table 8:** Minimum doses of miuraenamides and derivatives for anti-*Phytophthora* activity from Reference [49].

Compd	1	2	3	4	5	6	7	8	9	10
Dose [µg per disk]	0.025	0.025	0.025	1	10	0.13	5	2	>50	50

**Table 9:** Activity of crocacin analogues on NADH oxidase and vine downy mildew on plants.

Compd. 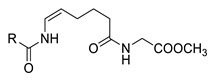	Side Chain R	IC_50_ NADH oxidAse (nM)	Breakpoint Vine Downy Mildew (µM)	T_50_ Glass Slide (h)
**81**	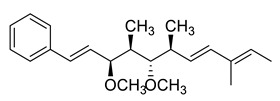	36	25	0.6
**82**	*n*-C_12_H_21_-	24	>100	0.1
**83**	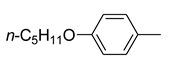	21	>100	5
**84**	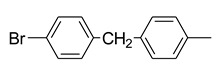	17	10	12
**85**	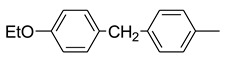	9	25	20
**86**	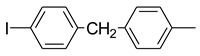	18	10	NT
**87**	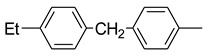	16	10	NT

**Table 10:** IC_50_ (µM) value of compounds **89**–**91** against porcine SCR. ^[a]^.

Cpd	R	IC_50_ (µM) ± SD
**89**	H	0.045 ± 0.001
**90a**	7-Br	0.012 ± 0.002
**90b**	6-Br	0.016 ± 0.001
**91**		0.70 ± 0.012
AZ ^[b]^		0.205 ± 0.001

^[a]^ from Reference [53,54]. ^[b]^ azoxystrobin.

**Table 11:** Inhibitory effect of selected compounds on SCR, SQR, and cyt bc1.

Cpd	IC_50_ (µM) SCR	I% (10 µM) SCR	I% (10 µM) SQR	I% (10 µM) cyt bc1	Selectivity
**103a**	3.55 ± 0.11	76%	14%	66%	cyt bc1
**103b**	9.39 ± 0.12	50%	<10%	46%	cyt bc1
**103c**	0.737 ± 0.011	82%	39%	83%	cyt bc1
**103d**	1.10 ± 0.20	73%	26%	67%	cyt bc1

**Table 12:** Comparative target site and in vitro antifungal activities of UK-2A and representative compounds **106**–**108** from References [61,62,63].

Compd	IC_50_ MET (nM)	EC_50_ (µg/L)
**UK-2A**	1.46	4.4
**106**	1.08	5.3
**107**	1.44	6.3
**108**	1.23	2.8
**AZ**	11.49	3.5

MET: Mitochondrial electron transport assays.
